# Switching positions: Assessing the dynamics of conjugational heterogeneity in antibody–drug conjugates using CE‐SDS

**DOI:** 10.1002/elps.202200140

**Published:** 2022-08-16

**Authors:** Eline B. A. van den Berg, Jaap C. W. Hendriks, Everdine W. Elsinga, Mark Eggink, Eef H. C. Dirksen

**Affiliations:** ^1^ Analytical Development and Quality Control (ADQC), Byondis B.V. Nijmegen The Netherlands; ^2^ Downstream Processing (DSP), Byondis B.V. Nijmegen The Netherlands

**Keywords:** antibody–drug conjugates, CE, conjugation positions, disulfide exchange, product characterization

## Abstract

Antibody–drug conjugates (ADCs) are a prospective class of new oncology therapeutics with the ability to deliver a cytotoxic drug to a targeted location. The concept appears simple, but ADCs are highly complex due to their intrinsic heterogeneity. Randomly conjugated ADCs, for instance, are composed of conjugated species carrying between 0 and 8 linker‐drug molecules, with several positional isomers that vary in drug distribution across the antibody. The drug load, expressed as drug‐to‐antibody ratio (DAR), is a critical quality attribute and should be well controlled, together with the distribution of drug molecules. Here, the impact of the duration of disulfide bond reduction on the DAR was investigated by quantitating the (isomeric) DAR species in ADCs produced with varying reduction times. Although hydrophobic interaction chromatography showed a constant DAR value as a function of reduction time, data obtained by non‐reducing CE‐SDS revealed an unexpected dynamic in the positional conjugated isomers. The insights obtained have improved our understanding of the correlation between the disulfide bond reduction, an important step in the manufacturing of a cysteine‐conjugated ADC, and the conjugational heterogeneity.

**Abbreviations**
(p)CQAs: (potential) critical quality attributes
ADC: antibody–drug conjugate
DAR: drug‐to‐antibody ratio
Fab: fragment antigen binding
HC: heavy chain
HH: heavy–heavy chains
HHL: heavy–heavy–light chains
HHLL: heavy–heavy–light–light chains (or intact mAb/ADC)
HIC: hydrophobic interaction chromatography
HL: heavy–light chains
LC: light chain
MeOH: methanol
MPA: mobile phase A
MPB: mobile phase B
nrCE‐SDS: non‐reduced capillary gel electrophoresis with sodium dodecyl sulfate
nrPEM: non‐reduced peptide mapping
QTPP: quality target product profile
TCEP: tris(2‐carboxyethyl)phosphine

## 1 INTRODUCTION

Antibody–drug conjugates (ADCs) comprise three key components: a monoclonal antibody (mAb) that binds to a specific antigen on the surface of the targeted cell, a (cleavable) linker molecule designed for covalent attachment and release of the drug following internalization of the ADC into the cell and a cytotoxic drug capable of inducing cell death after intracellular release and activation. ADCs are a prospective class of new oncology therapeutics with the ability to deliver cytotoxic small molecules to a targeted location (i.e., tumor cell). By using the specificity of the antibody moiety, drug effects are focused on the site of action, while undesired side effects of the cytotoxic molecule are minimized [1, 2, 3, 4]. Consequently, a fast‐expanding and competitive field has emerged in which several ADCs have been approved for commercial use, and many promising ADC candidates are being tested in clinical trials [5]. The principle of ADCs appears simple, but these compounds are in fact highly complex and heterogeneous. Following the successful development of so‐called first and second generation" ADCs, ongoing innovations aim to further improve the therapeutic window, potency, and/or selectivity. Several areas are being explored, like new payload mechanisms, increasing the drug load or tumor penetration, improving the efficiency of ADC uptake and cellular processing, or overcoming ADC resistance mechanisms [6]. In an attempt to improve therapeutic properties as well as the chemistry, manufacturing, and control (CMC) developability, site‐specific ADCs containing engineered cysteines have entered the stage. Linker‐drug conjugation can still be performed on cysteine residues, but without compromising existing disulfide bonds resulting in more homogeneous molecules [7, 8].

Initial ADC technologies, however, still prove to be effective with approved therapeutics such as *brentuximab vedotin* (Adcetris) for relapsed Hodgkin's lymphoma and systemic anaplastic large cell lymphoma and *ado‐trastuzumab emtansine* (Kadcyla) to treat HER2‐positive metastatic breast cancer. These “second‐generation” ADCs employ conjugation strategies where linker‐drug molecules are chemically linked to cysteine or lysine residues in the antibody [9]. Although the heterogeneity resulting from those conjugation strategies and complexity of these ADCs can be challenging, their design, development, and characterization have become more sophisticated over the years [10]. Consequently, an enhanced understanding of the behavior and physicochemical properties of ADCs throughout development has been obtained, which in turn aids in establishing correlations between product quality attributes and process parameters, ultimately leading to potential opportunities for the improvement of therapeutic properties of ADCs.

**FIGURE 1:**
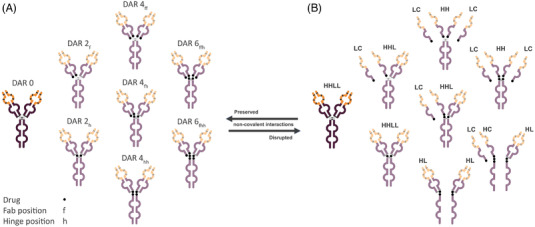
Illustration of the conjugational heterogeneity in a randomly cysteine‐conjugated antibody–drug conjugate (ADC) by variations in drug load (drug‐to‐antibody ratio [DAR]) and distribution (positional isomers). (A) Overview of intact DAR species, partially held together by non‐covalent interactions. (B) Overview of the constituents of each of the isomers, as formed under denaturing conditions

To initiate product and process development of an ADC, a quality target product profile (QTPP) is defined. The QTPP describes, amongst other things, the mechanism of action, envisioned indication, and characteristics important for the assessment of quality attributes impacting product safety and efficacy. The list of (potential) critical quality attributes ((p)CQAs) derived from the QTPP guides the development process of the ADC, together with relevant prior knowledge, to ensure quality of the product and its production process. One of the most important attributes of an ADC is the number of drug molecules linked to the antibody, expressed as the drug‐to‐antibody ratio (DAR) [11]. In the case of a randomly cysteine‐conjugated ADC, up to eight cysteine residues can be made available for linker‐drug conjugation in an IgG1‐type mAb. This is achieved by partially reducing the four interchain disulfide bonds connecting the heavy and light chains in the fragment binding antigen (Fab) domain, and both heavy chains in the hinge region [12]. The reduction of interchain disulfides does not, or hardly, affect the higher order structure of the antibody or its affinity to the antigen. The tertiary structure is maintained as a result of non‐covalent interactions between all chains [13, 14]. This reduction and conjugation strategy, however, results in a heterogeneous mixture of DAR species carrying up to (a maximum of) eight linker‐drugs *per* antibody. The resulting conjugational heterogeneity extends beyond these differences in drug load. DAR species with the same drug load are again composed of different individual species, defined as positional isomers, due to variations in the distribution of linker‐drug molecules across the antibody [15]. The qualitative and quantitative collection of (isomeric) DAR species, present in the ADC, eventually translates into an overall average DAR. In Figure 1, a schematical representation of the heterogeneity in DAR species and positional isomers in a randomly cysteine‐conjugated ADC is provided.

Together with protein concentration and purity, the average DAR determines the total amount of conjugated toxin delivered to the patient, which directly affects efficacy and cytotoxicity [16]. Therefore, the DAR is considered a CQA, as is the composition of DAR species [17]. Understanding the dynamics in the conjugational heterogeneity, and optimizing drug load and distribution in an ADC, is expected to improve the safety and physiochemical properties of ADC candidates [18]. Here, we set out to investigate the impact of the duration of disulfide bond reduction on the DAR and related heterogeneity of a randomly cysteine‐conjugated ADC.

The DAR of an ADC conjugated at the interchain cysteines is, amongst others, controlled by partial reduction of the antibody moiety. This step is affected by several parameters, such as the molar ratio of reducing agent (e.g., tris(2‐carboxyethyl)phosphine [TCEP]) to mAb and reaction conditions like pH, temperature, and reduction time. Improved understanding of this step could lead to a better prediction of the reduced interchain disulfides, thereby controlling the composition of DAR species. This results in an ADC with a consistent DAR and provides, if desired, the opportunity to reduce the heterogeneity in the ADC by the formation of specific positional isomers. The ADCs investigated were generated under different reduction conditions and subsequently analyzed using a set of physicochemical methods, to evaluate the correlation between the mAb reduction and conjugational heterogeneity.

The composition of DAR species that differs in drug load (Figure 1A) can be determined by hydrophobic interaction chromatography (HIC). HIC allows the separation of DAR species by differences in hydrophobicity as a result of varying numbers of hydrophobic linker‐drugs attached, while non‐covalent interactions and the tertiary structure are maintained under the relatively mild experimental conditions. This results in the quantitation of total amount of DAR species with the same drug load (i.e., sum of positional isomers). The resulting relative amounts of DAR can subsequently be used for the calculation of the overall average DAR.

To gain information about the positional isomers in the ADC, non‐covalent interactions must be disrupted to induce partial dissociation of the molecule (Figure 1B). As only a subset of cysteines involved in interchain disulfide bonds is reduced and conjugated, the dissociation of non‐covalently associated constituents allows for the identification of conjugated cysteines. Capillary gel electrophoresis with a non‐reducing and denaturing sample preparation with SDS (nrCE‐SDS) was selected for this purpose. This technique enables a robust and high‐resolution separation of ADC constituents following a disruption of non‐covalent interactions. In this way, the composition of positional isomers can be quantified. Following the observations made by nrCE‐SDS, non‐reducing peptide mapping (nrPEM) by liquid chromatography–tandem mass spectrometry (LC–MS/MS) analysis was performed to obtain further insight into the covalent disulfide bonds, as well as the conjugated cysteines present in the ADCs [19, 20].

## 2 MATERIALS AND METHODS

### 2.1 Materials

Multiple batches of the same ADC were produced in‐house at Byondis B.V. (Nijmegen, the Netherlands) using the same conditions, except for the reduction time of the mAb that varied between 0.5 and 24 hrs. Prior to analysis, ADC samples were purified by carbon filtration and HIC to remove non‐conjugated mAb and process‐related impurities. Finally, the ADCs were formulated in a buffer containing histidine, trehalose at pH 6 at a concentration of 10 mg/mL.

### 2.2 Methods

#### 2.2.1 Hydrophobic interaction chromatography (HIC‐HPLC)

HIC was performed using a TSKgel Butyl‐NPR column (3.5 cm × 4.6 mm, 2.5‐µm particle size, TOSOH, Joint analytical systems) on a Waters ACQUITY ARC (U)HPLCystem equipped with a PDA detector. Following a 5‐µL injection of 1‐mg/mL sample diluted in mobile phase A (MPA) (25‐mM phosphate, 1.5‐M ammonium sulfate at pH 6.95), components were eluted using a gradient of 0% – 100% mobile phase B (MPB) (25‐mM phosphate, pH 6.95 in 20% isopropanol) in MPA over 20 min at 27°C at a flowrate 0.4 mL/min. UV absorbance was detected at both 214 and 330 nm. Chromatograms were acquired and analyzed using the Empower chromatography data system (Waters).

#### 2.2.2 Non‐reduced capillary electrophoresis sodium dodecyl sulfate (nrCE‐SDS)

Capillary gel electrophoresis analysis was executed on a Sciex PA 800 Plus system with PDA detection employing a 50‐µm I.D. bare‐fused silica capillary (BGB) filled with a replaceable gel matrix (SDS‐MW gel buffer, Sciex). The equivalent of 75‐µg sample was diluted with water to a volume of 50 µL and mixed with 50‐µL sample buffer (25‐mM phosphate pH 6.6, 2.1% SDS (v/w), and 2% 10‐kDa internal standard (Sciex) (v/v)). Subsequently, the mixture was incubated at 80°C for 4 min at 300 rpm in a thermomixer (Eppendorf). Analysis was performed on the short‐end of a 50‐µm I.D. bare‐fused silica capillary trimmed to 30 cm (10‐cm effective capillary length) in a cartridge containing a 100 × 200‐µm aperture (Sciex). Prior to each sample analysis, the capillary was preconditioned with a 3‐min flush with 0.1‐M NaOH, 1‐min flush with 0.1‐M HCl, 1‐min flush with water, and a 10‐min flush with SDS‐MW gel to fill the capillary with fresh gel matrix. Separation of constituents was achieved after injecting the sample for 20 s with 5 kV and applying 15 kV on the capillary with 1379mbar simultaneous pressure at 25°C for 20 min. UV absorbance was detected at both 214 and 330 nm. Electropherograms were acquired and analyzed using the Empower chromatography data system (Waters).

#### 2.2.3 Non‐reduced peptide mapping using LC–MS/MS (nrPEM LC–MS/MS)

For nrPEM, a 100‐µg sample was denatured and alkylated for 1 hour in 8‐M urea with 6‐mM NEM at 30°C. The alkylated sample was digested for 2 hours with 2‐µg endoproteinase LysC at 30°C, followed by a 1‐hour digestion with 2‐µg trypsin in 50‐mM Tris–HCl, 0.6‐mM CaCl_2_ buffer at 30°C and finalized by the addition of 2‐µL TFA. A 10‐µl sample was injected onto an Ultimate 3000 UPLC system (Dionex, Thermo Scientific) equipped with an Acquity UPLC BEH300 C18 column (2.1 cm × 150 mm, 1.7‐µm particle size, Waters), a DAD detector and connected to an Orbitrap Fusion Tribrid mass spectrometer (Thermo Scientific). Peptides were eluted using a gradient increasing 0.1% formic acid (FA) in 80% ACN (MPB) from 0% to 80% in 0.1% FA (MPA) in 66 min at 60°C with a flowrate of 0.25 mL/min. Their UV absorbance was detected at 214, 280, and 330 nm, followed by ionization with an ESI source. MS data acquisition was done using Orbitrap detection with quadrupole isolation mode. MS was performed at positive polarity, Orbitrap resolution at 120 000 over a scan range *m*/*z* 400–1600. MS/MS was performed using electron‐transfer dissociation activation and an isolation window of 1.6 *m*/*z* over a normal scan range and Orbitrap resolution at 60 000. Data were analyzed by Xcalibur and PeptideFinder software (Thermo Scientific Corp.).

#### 2.2.4 Residual TCEP by LC–MS

For TCEP analysis, a sample was collected prior to conjugation and immediately filtered to remove the mAb. The analysis was executed on the Acquity UPLC HSS T3 column (2.1 cm × 100 mm, 1.8‐µm particle size, Waters) kept at 30°C with an Acquity UPLC system (Waters) connected to a MicrOTOF Q2 mass spectrometer (Bruker). Samples were diluted in MPA (0.1% FA in water) to a suitable level for quantitation, together with a limit reference preparation of 0.2 µg/ml. 5‐µl sample was injected and components were eluted with a gradient increasing MPB (0.1% FA in 50% ACN:MeOH) from 1% to 60% over 3 min with a flowrate of 0.2 ml/min. MS detection was done using ESI, and TCEP was detected via MS acquisition at full scan mode, 200–300 *m*/*z* range. The extracted ion current chromatograms were analyzed by QuantAnalysis (Bruker).

## 3 RESULTS AND DISCUSSION

To investigate the correlation between conditions employed to obtain a partially reduced mAb and the product quality of the resulting ADC, a criticality assessment was performed to identify reduction parameters that may impact product quality and/or consistency. Subsequently, any potentially critical parameter can be subjected to characterization studies to gain further insight into its individual impact according ICH Q9. Here, the reduction time was identified as one of the parameters influencing the DAR. Thereupon, a characterization study was conducted covering a wide range of reduction times, while keeping other conditions (e.g., amount of TCEP) constant, to enable the quantification of the impact by reduction time on DAR‐related attributes. ADCs were prepared in two consecutive experiments, one comprising ADCs produced with reduction times of 0.5, 1, 4, and 24 hours and a second experiment comprising reduction times of 1, 2, 3, and 4 hours. To evaluate experimental variation, ADCs were produced with reduction times of 1 and 4 hours in both experiments. Disulfide bond reduction was ended at each time point by proceeding to the conjugation step and attaching linker‐drug molecules to the available cysteines.

### 3.1 DAR analysis by HIC

**FIGURE 2:**
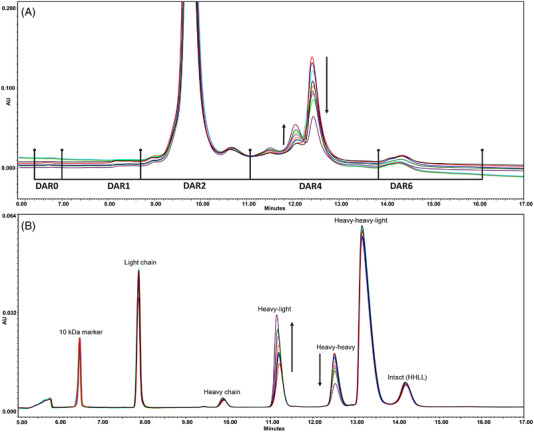
Results obtained for the antibody–drug conjugate (ADC) samples generated using varying reduction times. Samples are differentiated by experiment number (e.g., ADC.e01, ADC.e02) and reduction time (e.g., t0.5 h, t1 h): ADC GMP1 (black), ADC.e01/t0.5 h (red), ADC.e01/t1 h (dark blue), ADC.e02/t1 h (light blue), ADC.e02/t2 h (orange), ADC.e02/t3 h (pink), ADC.e02/t4 h (light green), ADC.e01/t4 h (dark green), and ADC.e01/t24 h (purple). (A) Overlay of hydrophobic interaction chromatography (HIC) chromatograms annotated with the drug‐to‐antibody ratio (DAR) species observed and the integration ranges used for their relative quantitation. The most prominent differences in DAR4‐related peaks are marked by arrows. (B) Overlay of non‐reduced capillary gel electrophoresis with SDS (nrCE‐SDS) electropherograms, annotated with the most abundant constituents observed. Those correlating to the DAR4 isomers are marked by arrows.

First, both sets of ADCs were analyzed using HIC to separate and quantify the DAR species by differences in drug load (e.g., DAR2, DAR4) according to the peak integration shown in Figure 2A and to determine the average DAR of each ADC. The resulting ADCs contained DAR species carrying up to six linker‐drug molecules *per* antibody. The results, presented in Table S1, show that the DAR remained unaffected by the duration of the reduction step. The relative amounts of DAR0, DAR1, DAR2, DAR4 and DAR6, and the average DAR did not differ considering the experimental variation of the replicate conditions, the composition observed in GMP batches produced at scale and the method precision. However, in contrast to the numerical results, differences were observed between the various ADC samples as a function of reduction time when overlaying the HIC chromatograms (Figure 2A). Therefore, the samples were further characterized by nrCE‐SDS to enable the identification and quantitation of the positional isomers within the DAR species.

### 3.2 Positional isomer characterization by nrCE‐SDS

Orthogonal analysis by nrCE‐SDS provided important insights into the conjugational heterogeneity of the ADCs produced at varying reduction times. The expected constituents (Figure 1B), light chain (LC), heavy chain (HC), heavy–light (HL) chains, heavy–heavy (HH) chains, heavy–heavy–light (HHL) chains, and heavy–heavy–light–light (HHLL) chains (or intact mAb/ADC), could be effectively resolved, along with the 10‐kDa internal standard. Evaluation of the electropherograms (Figure 2B) and resulting relative amounts of ADC constituents (see Table S2) revealed that the duration of the mAb reduction step influences the positional isomers formed. These dynamics are most clearly observed for the DAR4 species by the decrease in relative amount of conjugated HH, from 11.0% to 4.9% over time, accompanied by an increase of the conjugated HL constituent from 9.1% to 19.2%, respectively. This phenomenon extends to most conjugated DAR species comprising Fab (LC–HC) and hinge (HC–HC) positioned isomers, as evidenced by the decrease in relative amount of conjugated HHL species and concomitant increase in HHLL for the DAR2 isomers. A similar exchange of positional isomers occurs in DAR6 isomers. It shares the significant decrease in the HH constituent with the DAR4 isomers but is detected by the increase of HC constituents from 1.6% to 2.2%. The suggested changes in positional isomers are further supported by the decrease in the relative amount of conjugated LC from 20.3% to 16.7%. Conjugated LC is representative of Fab‐conjugated isomers, but not for hinge‐conjugated isomers (see Figure 1). The consistency in the number of disulfide bonds reduced is remarkable, given the dynamics in the overall population. Prolonged reduction times do not seem to increase the total number of cysteines available for conjugation, which would lead to a higher level of smaller constituents and increased average DAR. Rather, it generates new free cysteines while reoxidizing the initial cysteines to a covalent disulfide bond as evidence by a decrease in the relative amount of conjugated LC and increase in HL constituent (see Table S2), switching the conjugation position for the linker‐drug from the interchain cysteines located in the Fab domain to those located in the hinge region.

**FIGURE 3:**
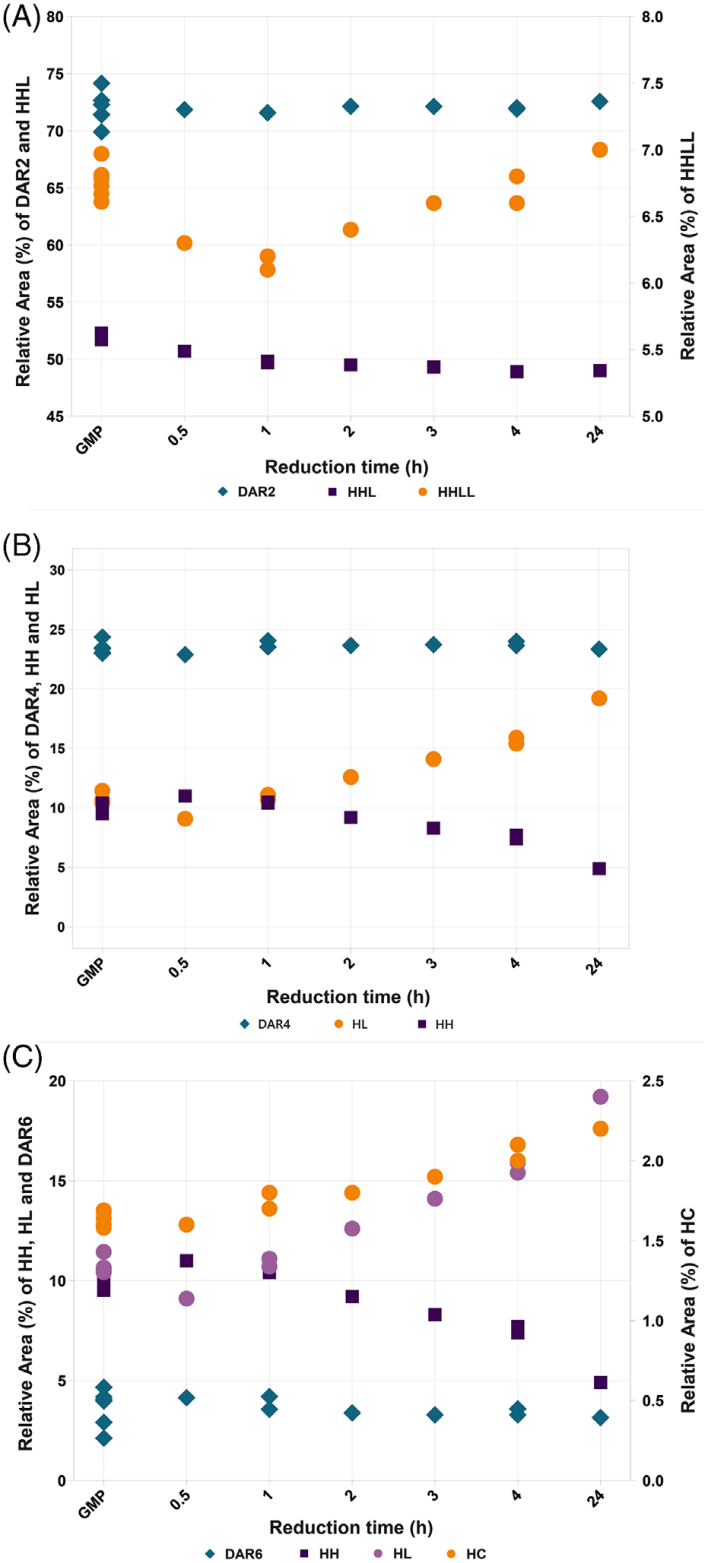
Drug‐to‐antibody ratio (DAR) species and antibody–drug conjugate (ADC) constituent data for the series of ADC samples obtained by hydrophobic Interaction chromatography (HIC) (diamonds) and non‐reduced capillary gel electrophoresis with SDS (nrCE‐SDS) (squares: Fab‐conjugated constituent, circles: hinge‐conjugated constituent(s)), together with data of GMP batches produced at scale. (A) Dynamics observed for the DAR2 species and its main constituents: heavy–heavy–light (HHL) and heavy–heavy–light–light (HHLL). (B) Dynamics observed for the DAR4 species and its main constituents: heavy–light (HL) and heavy–heavy (HH). (C) Dynamics observed for the DAR6 species and its main constituents: HH, HL, and heavy chain (HC). *Note*: *due to lower abundance of* *hinge‐conjugated isomers, the* *Y* *‐axis* *is split to enable the differences to be shown on one scale*.

This mechanism not only explains why the collection of DAR species quantitated by HIC was found to be unaffected by the reduction time, but also what is underlying the clear changes observed in the peak patterns for the different DAR species, as illustrated by the arrows marking the shift in DAR4 (Figure 2A). As positional isomers carry the same number of linker‐drug molecules, their hydrophobicity is highly similar hampering resolution by HIC. However, the heterogeneous peak patterns indicate that a subordinate separation of positional isomers is established based on differences in hydrophobicity. These distinctions occur when isomers are conjugated at different interchain positions, which in turn might impact the overall hydrophobicity of the molecule, resulting in retention differences on column. This effect is most prominent for DAR4, given the opposed nature of its Fab and hinge‐positioned isomers (see Figure 1). When evaluating the overlays of the HIC and nrCE‐SDS analyses presented in Figure 2, a correlation is observed between the shifting peak pattern of DAR4 on the one hand and the exchange between HH and HL on the other. The HIC and nrCE‐SDS data obtained for the constituents as a function of reduction time present in the ADCs generated here, as well as in a GMP batch manufactured at scale, are plotted in Figure 3. As can be seen in Figure 3, the relative amounts of DAR2, DAR4, and DAR6 remain constant, whereas their corresponding constituents display considerable shifts in relative amount, progressing from predominantly Fab‐conjugated to more hinge‐conjugated variants. The dynamics in the conjugational heterogeneity, as revealed by nrCE‐SDS, suggest that the cysteines available for conjugation change by subsequent reduction and reoxidation of the disulfide bonds over time. This was supported by free thiol analysis that confirmed all cysteines in the ADCs to either be involved in a disulfide bond or conjugated with a linker‐drug molecule at all reduction times (data not shown). Further characterization by nrPEM LC–MS/MS was performed to confirm the changes in positional isomers by quantifying the covalent disulfide bonds in the ADCs, as well as the conjugated cysteines. In addition, the amount of reductant (residual TCEP) was evaluated by RP–LC–MS to obtain information about the impact of this reagent on the reduction and reoxidation process of the disulfide bonds in the mAb.

### 3.3 Extended characterization of conjugated cysteines and disulfide bonds

**FIGURE 4:**
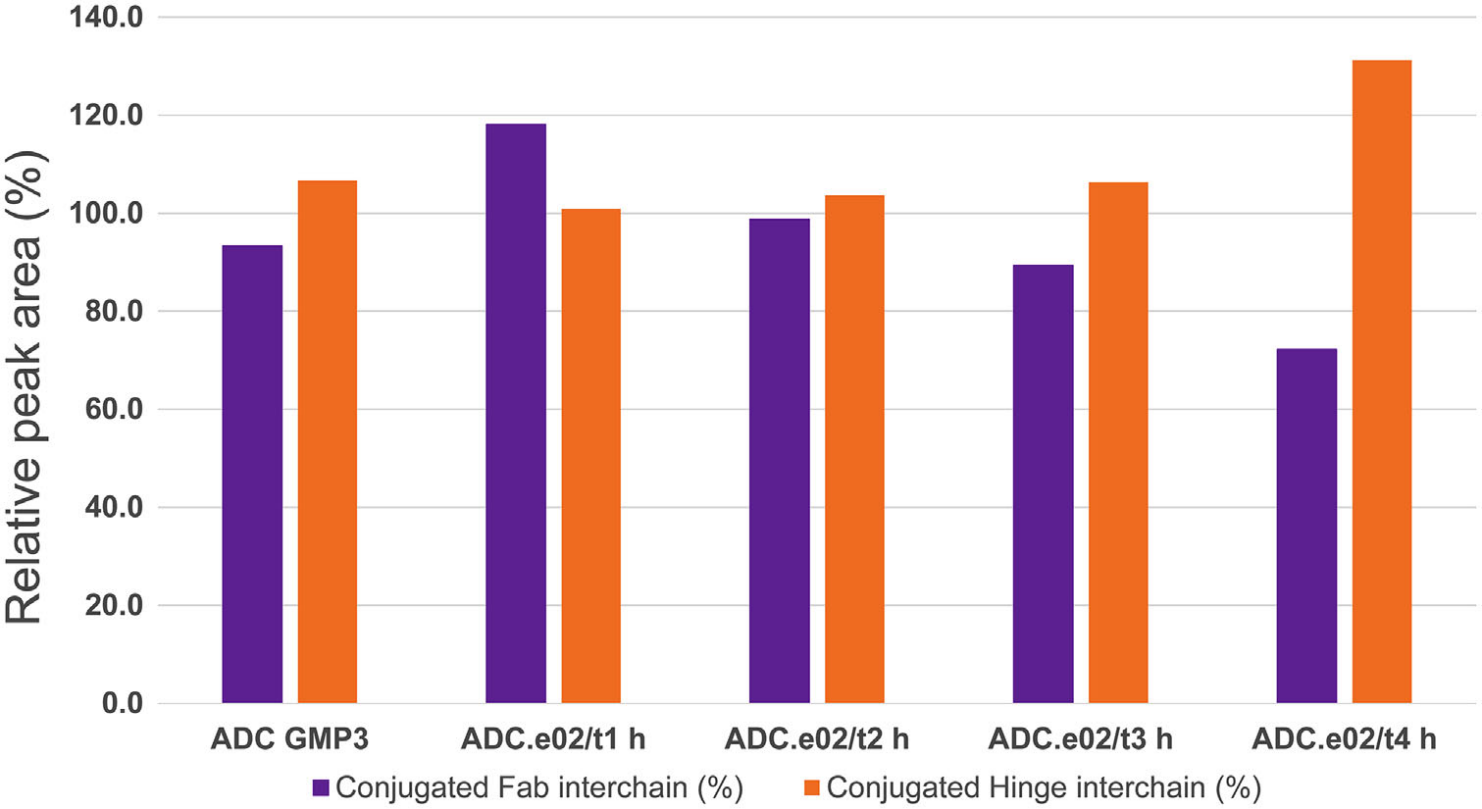
Conjugation distribution between Fab and hinge interchain positions in antibody–drug conjugates (ADCs) produced at reduction times of 1, 2, 3, and 4 hours and a GMP batch produced at scale. The values obtained by non‐reduced peptide mapping (nrPEM) LC–MS/MS are expressed as amount of Fab‐ or hinge‐conjugated peptides relative to the amounts detected in a representative reference standard (see Table S3).

nrPEM analysis not only confirmed the presence of all expected disulfide bonds in the ADCs, but also that prolonged reduction time did not induce disulfide bond scrambling (e.g., as a result of intrachain disulfide bond reduction). However, an increase in intensity of the disulfide‐linked HL dipeptide was observed as a function of reduction time, which supports the suggestion made with data obtained by nrCE‐SDS: reoxidation of cysteines located in the Fab region into covalent disulfide bonds. Furthermore, the data enabled the assessment of conjugated cysteines, confirming a shift in linker‐drug position by a decrease in the relative amount of conjugated peptides originating from the Fab domain, accompanied by an increase in conjugated hinge peptides, as can be seen in Figure 4.

Residual TCEP analysis by LC–MS revealed no significant amounts of, or differences in, residual TCEP in any of the ADC samples. This indicates that the total amount of TCEP added reacts within the first 0.5 hr of the mAb reduction (Table S4). Therefore, it is unlikely that TCEP initiates the continued reduction of the hinge disulfides over time. This correlates with the nrCE‐SDS data, which revealed no increase of smaller mAb constituents, but merely a variation in the location of the reduced disulfide bonds.

**FIGURE 5:**
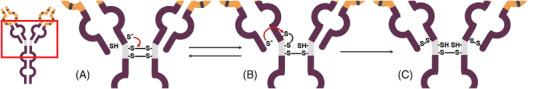
Proposed mechanism for the thiol‐disulfide exchange occurring within a mAb over the course of the partial reduction time. (A) A deprotonated thiol (S‐) displaces the sulfur of a hinge‐located disulfide bond, resulting in a mixed‐disulfide between Fab and hinge cysteines. (B) In the subsequent exchange reaction, the new thiolate in the Fab attacks the mixed‐disulfide bond resulting in reoxidation of the disulfide in the Fab region and reduction of a hinge disulfide bond, switching the available cysteine for conjugation (C).

The nrCE‐SDS data, presented in Table S2 and Figure 3, reveal a predominance of Fab interchain‐conjugated species at relatively short reduction times, which has been reported to be the preferred conjugation position for an IgG1 [11]. Nevertheless, over time, reduced disulfide bonds within the Fab region switch to those located in the hinge, as evidenced by the changes observed in linker‐drug position in the ADCs. The specific mechanism behind this positional switching is not fully understood (yet). The ability to form and break a disulfide bond depends on several factors. A disulfide bond can be broken by a reductant but is reversible under appropriate conditions. As described by Sevier et al., a reaction occurring in proteins is an intramolecular thiol‐disulfide exchange [21]. In this reaction, electrons are exchanged between a cysteine and a disulfide bond and serves to oxidize a thiol (R_1_SH) while reducing a disulfide (R_2_SSR_2_). A schematic of the proposed mechanism occurring in the ADCs investigated here as a function of reduction time is presented in Figure 5.

The thiol‐redox reaction between the free cysteines in the Fab domain and those involved in the hinge disulfide bonds may be initiated at prolonged reduction times, suggesting that the flexibility of the hinge region and the position of the cysteines therein are important for restoring (interchain) disulfide bonds within the antibody, thereby maintaining the higher order structural integrity of the molecule [7, 21–23]. The thiol‐disulfide reaction appears to proceed until 24 h. Considering the overall duration of an ADC manufacturing process, no ADCs were generated at reduction times beyond 24 h. Therefore, it remains unclear if longer reduction times would result in fully hinge‐conjugated ADCs. The hypothesized thiol‐disulfide reaction and the effect of reduction times beyond 24 hours could be investigated in follow‐up experiments.

### 3.4 Annotation of the HIC peak profile

In addition to the improved understanding of the impact of reduction time on the DAR‐related heterogeneity, the information reported here improved the interpretation and annotation of HIC chromatograms as well. By combining the HIC and nrCE‐SDS data, the most abundant DAR4 peak in the HIC chromatogram could be annotated as isomer DAR4ff (see Figure 1), whereas the second highest DAR4 peak, eluting at an earlier retention time, was annotated as isomer DAR4hh. As suspected, a hinge interchain‐conjugated positional isomer is less hydrophobic as a result of the more shielded linker‐drug molecules. Similar observations were made for DAR6 as function of reduction time. A decrease in the relative amount of the most abundant DAR6 peak, corresponding to a decrease of the HH constituent in nrCE‐SDS, annotates this peak as isomer DAR6ffh, while at the same time the relative amount of HC constituent, the representative of positional isomer DAR6fhh, increases. Likewise, the main DAR2 peak was confirmed to comprise two co‐eluting positional isomers, DAR2f and DAR2h, with the detection of HHL and HHLL constituents. Just like the DAR4, DAR2f is found to be the preferred positional isomer at relatively short reduction time.

## 4 CONCLUDING REMARKS

The conjugational heterogeneity of cysteine‐conjugated ADCs is a complex interplay between DAR species on one hand and positional isomers on the other. This heterogeneity is influenced by several parameters. To better understand how these parameters affect the DAR‐related heterogeneity, an investigation was performed to determine the impact of the reduction time on the qualitative and quantitative collection of (isomeric) DAR species.

The collection of DAR species was found to remain unaffected by the reduction time, just like the resulting average DAR. Further investigation of the HIC chromatograms of the various ADC samples indicated a variation within the composition of individual DAR species, most prominently in DAR4. To gain information regarding these differences in conjugational heterogeneity, nrCE‐SDS analysis was performed, which revealed that the reduction time influences the dynamics in the population of positional isomers, as evidenced by the composition of constituents shifting from predominantly Fab‐conjugated to more hinge‐conjugated variants. A prolonged reduction time was found not to increase the total amount of free cysteines available for conjugation, resulting in higher levels of smaller constituents and increased DAR. Rather, it generates new free cysteines while reoxidizing the initial cysteines into a covalent disulfide bond, switching linker‐drug conjugation from the interchain cysteines in the Fab domain to the disulfide bonds of the hinge region (see Figures 3 and 4).

This unexpected observation emphasizes the importance of CE in the analytical toolbox for the characterization of randomly cysteine‐conjugated ADCs. Here, the added value of nrCE‐SDS was shown with the quantitation of the positional isomeric “blind spots” of HIC, while conversely, HIC allowed for the quantification of the intact DAR species formed by the ADC constituents. The nrCE‐SDS method employed in this characterization study was validated and showed to enable accurate (99%–102%), linear (>0.99), and precise (<2.0%) quantitation of each individual ADC constituent, as well as overall purity. nrCE‐SDS was found to be an excellent complement to HIC and alternative for RP‐HPLC, which is routinely used for the purity evaluation and heterogeneity analysis of ADCs, making it a fast and easy substitute assay for monitoring purity, the conjugation positions, and consistency therein.

## CONFLICT OF INTEREST

The authors have declared no conflict of interest.

## Supporting information

Supporting Information

## Data Availability

The data that support the findings of this study are available on request from the corresponding author. The data are not publicly available due to confidentiality restrictions.
