# Supplementary material for: Switching positions: Assessing the dynamics of conjugational heterogeneity in antibody–drug conjugates using CE‐SDS
Source: Electrophoresis. 2022 Aug 16;44(1-2):62–71. doi: 10.1002/elps.202200140 (PMC10086850; doi:10.1002/elps.202200140)
Supplement: Supplementary file 1 — Supporting Information [file ELPS-44-62-s001.docx]

Supporting Information

*Table 1: The relative amounts of DAR0, DAR1, DAR2, DAR4, DAR6 and average DAR values determined by HIC for the series of ADC samples generated using varying reduction times together with the DAR composition of two GMP batches produced at scale and analytical assay variation determined in method performance trending (expressed as +/- 3σ of the run-to-run variance for the reportable value). The batch codes are differentiated by experiment number (e.g., ADC.e01, ADC.e02) and reduction time (e.g., t0.5 h, t1 h).*

| Batch | Reduction time (h) | DAR0 (%) | DAR1 (%) | DAR2 (%) | DAR4 (%) | DAR6 (%) | Average DAR |
| --- | --- | --- | --- | --- | --- | --- | --- |
| Analytical variation | N/A | 0.1 | 0.3 | 0.9 | 0.3 | 0.7 | 0.03 |
| ADC GMP1 | 01:28 | 0.2 | 1.1 | 71.2 | 23.6 | 4.0 | 2.6 |
| ADC GMP2 | 01:22 | 0.1 | 0.6 | 72.3 | 23.1 | 4.0 | 2.6 |
| ADC.e01/t0.5 h | 00:31 | 0.3 | 0.8 | 71.8 | 22.9 | 4.1 | 2.6 |
| ADC.e01/t1 h | 01:07 | 0.2 | 0.5 | 71.6 | 23.5 | 4.2 | 2.6 |
| ADC.e02/t1 h | 01:03 | 0.4 | 0.4 | 71.6 | 24.1 | 3.6 | 2.6 |
| ADC.e02/t2 h | 02:05 | 0.3 | 0.5 | 72.1 | 23.7 | 3.4 | 2.6 |
| ADC.e02/t3 h | 03:06 | 0.3 | 0.6 | 72.1 | 23.7 | 3.3 | 2.6 |
| ADC.e02/t4 h | 04:06 | 0.3 | 0.5 | 71.9 | 24.0 | 3.3 | 2.6 |
| ADC.e01/t4 h | 04:13 | 0.2 | 0.5 | 72.0 | 23.7 | 3.6 | 2.6 |
| ADC.e01/t24 h | 24:11 | 0.3 | 0.7 | 72.6 | 23.3 | 3.2 | 2.6 |

Table 2: The relative amounts of each constituent (LC, HC, HL, HH, HHL, HHLL) by nrCE-SDS for the series of ADC samples generated using varying reduction times together with the constituent composition of two GMP batches produced at scale and analytical assay variation determined in method performance trending (expressed as +/- 3σ of the run-to-run variance for the reportable value). The batch codes are differentiated by experiment number (e.g., ADC.e01, ADC.e02) and reduction time (e.g., t0.5 h, t1 h).

| Batch | Reduction time (h) | LC (%) | HC (%) | HL (%) | HH (%) | HHL (%) | HHLL (%) |
| --- | --- | --- | --- | --- | --- | --- | --- |
| Analytical variation | N/A | 0.7 | 0.1 | 0.1 | 0.1 | 0.6 | 0.2 |
| ADC GMP1 | 01:28 | 19.2 | 1.7 | 10.4 | 10.2 | 51.0 | 6.5 |
| ADC GMP2 | 01:22 | 18.6 | 1.7 | 10.7 | 10.3 | 51.1 | 6.6 |
| ADC.e01/t0.5 h | 00:31 | 20.3 | 1.6 | 9.1 | 11.0 | 50.7 | 6.3 |
| ADC.e01/t1 h | 01:07 | 19.9 | 1.8 | 11.1 | 10.4 | 49.7 | 6.2 |
| ADC.e02/t1 h | 01:03 | 20.2 | 1.7 | 10.7 | 10.5 | 49.8 | 6.1 |
| ADC.e02/t2 h | 02:05 | 19.4 | 1.8 | 12.6 | 9.2 | 49.5 | 6.4 |
| ADC.e02/t3 h | 03:06 | 18.8 | 1.9 | 14.1 | 8.3 | 49.3 | 6.6 |
| ADC.e02/t4 h | 04:06 | 18.4 | 2.0 | 15.4 | 7.7 | 48.9 | 6.6 |
| ADC.e01/t4 h | 04:13 | 18.0 | 2.1 | 15.9 | 7.4 | 48.9 | 6.8 |
| ADC.e01/t24 h | 24:11 | 16.7 | 2.2 | 19.2 | 4.9 | 49.0 | 7.0 |

Table 3: The relative amounts of Fab and hinge conjugation by nrPEM for the series of ADC samples generated using varying reduction times together with the expected conjugation positions of a GMP batch produced at scale. The values are expressed as amount of Fab- or hinge-conjugated peptides relative to the amounts detected in a representative ADC batch taken along as reference standard. The batch codes are differentiated by experiment number (e.g., ADC.e01, ADC.e02) and reduction time (e.g., t0.5 h, t1 h).

| Batch | Reduction time (h) | Conjugated Fab interchain (%) | Conjugated Hinge interchain (%) |
| --- | --- | --- | --- |
| ADC GMP3 | 01:25 | 93.3 | 106.6 |
| ADC.e02/t1 h | 01:03 | 118.1 | 100.9 |
| ADC.e02/t2 h | 02:05 | 98.8 | 103.6 |
| ADC.e02/t3 h | 03:06 | 89.3 | 106.4 |
| ADC.e02/t4 h | 04:06 | 72.2 | 131.2 |

Table 4: The TCEP concentration during ADC production at 0, 0.5, 1, 4 and 24 hours together with the used reduction buffer containing the starting concentration TCEP. The batch codes are differentiated by experiment number (e.g., ADC.e01, ADC.e02) and reduction time (e.g., t0.5 h, t1 h).

| Batch | Reduction time (h) | TCEP (µg/mL) |
| --- | --- | --- |
| Reduction buffer | N/A | 141.5 |
| ADC.e01/t0 h | 00:00 | 1.5 |
| ADC.e01/t0.5 h | 00:31 | <0.2 |
| ADC.e01/t1 h | 01:07 | <0.2 |
| ADC.e01/t4 h | 04:13 | <0.2 |
| ADC.e01/t24 h | 24:11 | <0.2 |
